# Microbiome and metabolome analyses of milk and feces from dairy cows with healthy, subclinical, and clinical mastitis

**DOI:** 10.3389/fmicb.2024.1374911

**Published:** 2024-06-06

**Authors:** Chenglin Zhu, Yuxuan Zhao, Falong Yang, Qian Zhang, Xin Zhao, Zhibo Yang, Xiaofang Dao, Luca Laghi

**Affiliations:** ^1^College of Food Science and Technology, Southwest Minzu University, Chengdu, China; ^2^College of Animal and Veterinary Sciences, Southwest Minzu University, Chengdu, China; ^3^Department of Agricultural and Food Sciences, University of Bologna, Cesena, Italy

**Keywords:** dairy cows, mastitis, milk, feces, microbiome, metabolome

## Abstract

Mastitis is commonly recognized as a localized inflammatory udder disease induced by the infiltration of exogenous pathogens. In the present study, our objective was to discern fecal and milk variations in both microbiota composition and metabolite profiles among three distinct groups of cows: healthy cows, cows with subclinical mastitis and cows with clinical mastitis. The fecal microbial community of cows with clinical mastitis was significantly less rich and diverse than the one harbored by healthy cows. In parallel, mastitis caused a strong disturbance in milk microbiota. Metabolomic profiles showed that eleven and twenty-eight molecules exhibited significant differences among the three groups in feces and milk, respectively. Similarly, to microbiota profile, milk metabolome was affected by mastitis more extensively than fecal metabolome, with particular reference to amino acids and sugars. Pathway analysis revealed that amino acids metabolism and energy metabolism could be considered as the main pathways altered by mastitis. These findings underscore the notable distinctions of fecal and milk samples among groups, from microbiome and metabolomic points of view. This observation stands to enhance our comprehension of mastitis in dairy cows.

## 1 Introduction

Mastitis is the disease that most often causes inflammation of cows’ mammary glands, due to the infection by various pathogenic bacteria, which leads serious problems to dairy cows’ health and, in turn, profitability of dairy farms (Ezzat Alnakip et al., 2014). Moreover, the excessive utilization of veterinary drugs for mastitis treatment could potentially engender a looming concern for human health, given the potential connection to antibiotic residues. An exceptionally critical facet in the battle against mastitis lies in the potential to achieve prompt, dependable, and precise disease diagnosis. In fact, mastitis, when subclinical, could spread unnoticed within a herd, resulting in more losses than clinical mastitis (Kaczorowski et al., 2022). Till now, the California mastitis test (CMT) and somatic cell count (SCC) are widely accepted as convenient and rapid methods to diagnose subclinical mastitis. However, several noninfectious factors can affect SCC, in turn leading to falsely positive results (Sargeant et al., 2001). Hence, the desirability of incorporating supplementary diagnostic methods emerges to augment the precision of cow mastitis detection. Metabolomics perfectly fulfill the above requirements, which can provide a platform for biomarker discovery and pathway identification of diseases. In our recent research endeavors focused on investigating the impact of clinical mastitis on cow biofluids, we have effectively employed ^1^H-NMR-based metabolomics to comprehensively analyze the metabolic distinctions between milk samples obtained from healthy cows and those afflicted with clinical mastitis (Zhu et al., 2021). Through the integration of findings from fecal, serum, and urinary analyses, it becomes evident that clinical mastitis exerts an influence on various pathways associated with energy metabolism and amino acid metabolism (Zhu et al., 2023).

Although previous studies by analysis of metabolic fingerprints have already rendered promising results, in terms of biomarkers identification and pathways elucidation, there is a growing recognition that the gut microbiota could potentially assume a pivotal role in upholding the physiological homeostasis of the host (Ma et al., 2018). Specifically, the entero-mammary pathway hypothesis has delineated a potential correlation between certain indigenous gut bacteria and the onset and progression of mastitis (Hu et al., 2020). In this context, Zhao et al. demonstrated that gut dysbiosis contributes to the pathogenesis of mastitis through the augmentation of bacterial translocation and systemic inflammation. In addition, administration of commensal *Roseburia* to mice was found to alleviate gut-dysbiosis-induced mastitis, by means of butyrate production, associated to barrier repair and reduced inflammation (Zhao et al., 2022).

To the best of our knowledge, the parallel analysis of metabolome and microbiome of feces and milk has only been conducted in a few studies (Wang et al., 2020; Hu et al., 2021a). Furthermore, the specific mechanism of mastitis remains not entirely clear. To examine the gut inner environment (microbiota and metabolites) and inflammatory reaction during mastitis, we collected feces and milk samples from healthy, subclinically mastitic and clinically mastitic cows. Then, we used 16S rRNA gene sequencing and untargeted metabolomics to analyze the profile of microbiota and metabolites in both feces and milk. These data provide further understanding of the correlations between gut and milk microbial community and metabolite profiles of dairy cows with differing udder health status, as well as a new perspective for the early diagnosis of dairy cows with subclinical mastitis.

## 2 Materials and methods

### 2.1 Animals and samples

All experimental designs and protocols were approved by the Animal Ethics Committee of Southwest Minzu University (Chengdu, China; approval number: SWUN-A-0050) and were in accordance with the recommendations of the academy’s guidelines for animal research.

The study was conducted during the period from July to August 2022. Chinese Holstein cows (parity: 2–4, days in milk: 150–195 days) were carefully chosen from a meticulously maintained large-scale dairy farm situated in the outskirts of Jiangsu, China. These cows were accommodated within free housing systems and were provided with total mixed rations (TMR) in adherence to established norms during their indoor tenure.

In the present study, the udder status of cows was comprehensively judged according to the degree of inflammation in each quarter, based on the clinical manifestations and on the results of the CMT and SCC in milk. Following the suggestions of Wang et al. (2022b) and according to the records, 30 Chinese Holstein cows were selected and divided into 3 groups: the H group was constituted by ten cows with healthy udder (SCC <100,000 cells/mL; no clinical symptoms of inflammation in udders; negative CMT results); the S group comprised ten cows with subclinical mastitis (500,000 < SCC < 800,000 cells/mL, no obvious clinical symptoms of inflammation in the udders, weakly positive CMT results); the C group was made by ten cows with clinical mastitis (SCC > 1,000,000 cells/mL; obvious signs of inflammation in the udders, including udder swelling, redness and milk clots, etc.; positive or strongly positive CMT results). Along the present work, the fecal samples from the three groups will be identified with FH, FS, and FC, while the corresponding milk samples will be identified by MH, MS, and MC. Milking procedures were conducted thrice daily through the utilization of an automated milking system (Ruishengyuan Machinery Assembly Co., Ltd., Hengshui, Hebei, China). To prevent the spread of inflammation through the entire dairy herd, cows designated as H, SM, and CM were housed in 3 separate cowsheds. None of the cows received treatments with antibiotics or other drugs. A total of 15 mL of composite milk was extracted in a sterile centrifuge tube from each animal, with roughly equivalent volumes obtained from each lactating udder quarter. For fecal sampling, sterile gloves were employed to collect rectal samples from each cow, with the initial few fecal streams being discarded prior to actual sample collection. Sampled feces were collected into sterile airtight bags, which were placed in a foam box with ice packs. Following the collection of fecal and milk samples, all specimens were promptly placed under refrigeration with ice to facilitate their transportation to the laboratory within a 2 h timeframe.

### 2.2 Metabolome analysis

The preparation of fecal and milk samples for ^1^H-NMR analysis followed the methods outlined by Zhu et al. (2021) and Zhang et al. (2024). Briefly, fecal samples were processed for ^1^H-NMR analysis by vortex mixing 80 mg of stool with 1 mL of deionized water for 5 min. The resulting mixtures were subsequently centrifuged at 18,630 g and 4°C for 15 min, and 0.7 mL of the supernatant was combined with 0.2 mL of NMR analysis solution. This solution consisted of D_2_O containing 10 mmol/L of 3-(trimethylsilyl)-propionic-2,2,3,3-d4 acid sodium salt (TSP) as the NMR chemical-shift reference, and 2 mmol/L of NaN_3_ to inhibit microbial growth. The pH of the solution was buffered at 7.00 ± 0.02 using 1 mol/L phosphate buffer.

For milk samples, 0.7 mL was mixed with 0.8 mL of CHCl_3_, then vortexed for 3 min and centrifuged at 18,630 g and 4°C for 15 min to remove fat. Afterward, 0.5 mL of the supernatant was combined with 0.2 mL of the NMR analysis solution. Each sample underwent another round of centrifugation under the aforementioned conditions.

The ^1^H-NMR spectra were acquired at 298 K using an AVANCE III spectrometer (Bruker, Wuhan, China) operating at a frequency of 600.13 MHz. Following the approach by Zhu et al. (2020), the suppression of signals from broad resonances originating from large molecules was accomplished using a CPMG-filter consisting of 400 echoes with a *τ* of 400 μs and a 180° pulse of 24 μs, resulting in a total filter duration of 330 ms. The residual signal from HOD was suppressed through presaturation using the cpmgpr1d sequence from the standard pulse sequence library.

Each spectrum was acquired by summing 256 transients with 32 K data points over a 7,184 Hz spectral window, employing an acquisition time of 2.28 s and a recycle delay of 5 s. Baseline adjustment of the ^1^H-NMR spectra was conducted using peak detection following the “rolling ball” principle (Kneen and Annegarn, 1996) and implemented using the baseline R package (Liland et al., 2010). To account for differences in water and fiber content among samples, probabilistic quotient normalization was applied to the entire spectrum array according to Dieterle et al. (2006). Signal assignments were made by comparing their chemical shift and multiplicity with the Chenomx software library (Chenomx Inc., Canada, ver 8.4), following the methodology of Zhu et al. (2020).

### 2.3 Microbiota analysis

Total microbial genomic DNA was extracted from fecal and milk samples using the FastDNA^®^ Spin Kit for Soil (MP Biomedicals, United States) according to manufacturer’s instructions. The quality and concentration of DNA were determined by 1.0% agarose gel electrophoresis and a NanoDrop2000 spectrophotometer (Thermo Scientific, United States) and kept at −80°C prior to further use. This DNA served as the template for PCR amplification of bacterial 16S rRNA genes utilizing barcoded primers and Takara Ex Taq (Takara, China). The hypervariable region V3–V4 of the bacterial 16S rRNA gene were amplified with primer pairs 343F (5’-TACGGRAGGCAGCAG-3′) and 798R(5’-AGGGTATCTAATCCT-3′) by T100 Thermal Cycler PCR thermocycler (BIO-RAD, United States).

The PCR product was extracted from 2% agarose gel and purified using the PCR Clean-Up Kit according to manufacturer’s instructions and quantified using Qubit 4.0 (Thermo Fisher Scientific, United States). Purified amplicons were pooled in equimolar amounts and paired-end sequenced on an Illumina PE300 platform (Illumina, United States) according to the standard protocols.

Raw sequencing data was obtained in FASTQ format. Paired-end reads underwent initial preprocessing using the cutadapt software to identify and remove adapter sequences. Following adapter trimming, paired-end reads underwent further processing to eliminate low-quality sequences, denoising, merging, and detection of chimera reads using DADA2 within the QIIME2 (2020.11) framework (Callahan et al., 2016). This culminated in the acquisition of representative reads and an abundance table for amplicon sequence variants (ASVs). The selection of a representative read for each ASV was accomplished using the QIIME2 package (Bolyen et al., 2019). Subsequently, all representative reads underwent annotation and BLAST-based comparisons against the Silva database, Version 138 (16S rDNA) using the q2-feature-classifier with default parameters. The raw sequencing reads were deposited into the NCBI Sequence Read Archive (SRA) database (Accession Number: PRJNA1095683).

### 2.4 Statistical analysis

Statistical analysis was executed using the R computational language. ANOVA followed by the Tukey HSD test was employed to ascertain statistically significant variations in microbiota and molecules across the three groups. A significance threshold of *p* < 0.05 was adopted for this purpose. In instances where variables exhibited non-normal distribution, transformations were applied according to the methodology outlined by Box and Cox (1964).

To detect differences in microbial abundance among distinct groups and discern the contribution of the microbiota profile to these differences, Linear Discriminant Analysis Effect Size (LEfSe) and Linear Discriminant Analysis (LDA) were performed. These analyses were carried out using the Oebiotech Cloud Platform.^1^

To elucidate the underlying trends within the metabolome of the samples, a robust Principal Component Analysis (rPCA) model was constructed (Hubert et al., 2005), focusing on the molecules identified through the aforementioned univariate analysis. The model encompassed a score plot, visualizing sample projection within the PC space, designed to emphasize the inherent data structure. Additionally, a Pearson correlation plot was generated, correlating the concentration of each molecule with the model’s components.

Pathway enrichment analysis was conducted using MetaboAnalyst 5.0 (Chong et al., 2018) to identify pertinent pathways by amalgamating the results obtained from the analysis. This approach concentrated on molecules that demonstrated significant concentration variations as per the univariate analysis.

To investigate correlations between fecal microbiota and metabolites in both feces and milk, Spearman correlation analysis was performed using the aforementioned online tool.

## 3 Results

### 3.1 Fecal and milk microbiota among cows in different groups

A total of 15,860 ASV were obtained from the fecal and milk samples analyzed. The *α*-diversity of fecal and milk microbiota among the three groups showed significant differences, as shown in Tables 1, 2, respectively.

**Table 1:** *α*-diversity indices of fecal bacteria.

Items	FH (*n* = 10)	FS (*n* = 10)	FC (*n* = 10)	SEM*	*p*-value
Simpson	0.99^a^	0.98^a^	0.95^a^	9.43 × 10^−3^	0.101
Chao1	919.05^a^	774.05^b^	497.88^c^	35.88	<0.001
Observed species	903.46^a^	760.75^b^	489.76^c^	35.15	<0.001
Shannon	8.59^a^	8.08^a^	7.02^b^	0.17	<0.001
Goods coverage	0.99^b^	0.99^b^	1.00^a^	1.24 × 10^−4^	0.002
PD whole tree	33.87^a^	29.41^b^	22.45^c^	1.05	<0.001
ACE	916.70^a^	772.48^b^	495.46^c^	35.87	<0.001

*SEM stands for standard error of mean. Within a row, different letters mean that values differed significantly (*p* < 0.05).

**Table 2:** *α*-diversity indices of milk bacteria.

Items	MH (*n* = 10)	MS (*n* = 10)	MC (*n* = 10)	SEM*	*p*-value
Simpson	0.98^a^	0.97^b^	0.96^c^	2.20 × 10^−3^	<0.001
Chao1	629.738^a^	530.84^a^	434.35^b^	21.09	<0.001
Observed species	624.42^a^	525.55^a^	431.47^b^	20.92	<0.001
Shannon	7.88^a^	7.45^b^	6.90^c^	0.10	<0.001
Goods coverage	0.99^b^	0.99^b^	1.00^a^	1.93 × 10^−5^	<0.001
PD whole tree	26.78^b^	22.59^ab^	20.74^b^	0.83	<0.001
ACE	629.86^a^	529.13^a^	433.15^b^	21.05	<0.001

*SEM stands for standard error of mean. Within a row, different letters mean that values differed significantly (*p* < 0.05).

Referring to fecal samples, as for community richness, compared with the FH group, FS and FC groups showed lower ACE and observed species indexes. As for community diversity, Shannon indexes were significantly lower in the FC group compared with FH and FS groups. According to the number of ASV sampled, rarefaction curves were obtained, which leveled off as the number of tags increased, indicating sufficient sequencing depth (Supplementary Figure S1). Similar to fecal samples, as for community richness, MC samples showed lower values of ACE and observed species compared to MH and MS samples. As for community diversity, Simpson and Shannon indexes were significantly lower in the MS and MC samples, compared to MH. According to the number of ASV sampled, rarefaction curves were obtained, which leveled off as the number of tags increased, indicating sufficient sequencing depth (Supplementary Figure S2).

**Figure 1:**
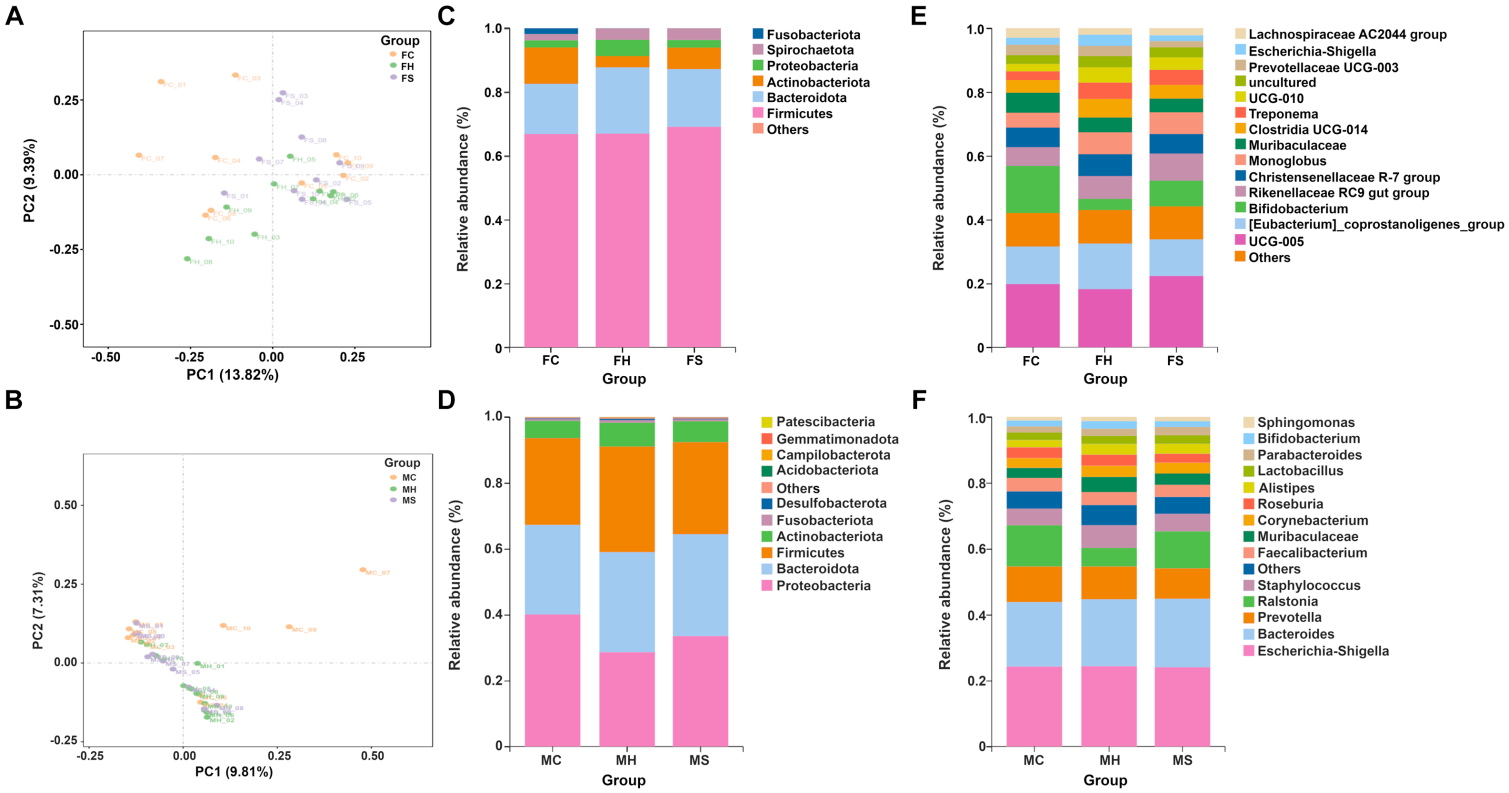
The *β*-diversity and microbiota compositions of fecal and milk among groups separately (*n* = 10). **(A)** PCA analysis for feces; **(B)** PCA analysis for milk; **(C)** Microbiota composition of feces at phyla level; **(D)** Microbiota composition of milk at phyla level; **(E)** Microbiota composition of feces at genus level; **(F)** Microbiota composition of feces at genus level.

The difference of fecal and milk microbial profiles among groups were initially distinguished using *β*-diversity analysis, as shown in Figures 1A,B.

As shown in Figure 1A, PCA analysis showed spatial separation in fecal microbiota among FH, FS, and FC groups. The distances between the FC and FH samples were greater than those between the FS and FH samples, indicating greater differences in fecal microorganism profiles between cows healthy and with clinical mastitis. In terms of milk samples, as depicted in Figure 1B, PCA analysis showed spatial separation in milk microbiota among MH, MS, and MC groups. The distances between the MC and MH group samples were greater than those between the MS and MH groups, indicating greater differences in milk microorganism profiles between cows with clinical mastitis and healthy.

Taxonomic annotation on ASV was conducted to identify the composition and relative abundance of fecal and milk microbiota. As shown in Figures 1C,D, *Firmicutes*, *Bacteroidota*, *Actinobacteriota* and *Proteobacteria* had the highest abundance in both fecal and milk samples. At the genus level, 136 genera were detected in all fecal microbiota, in which *UCG-005*, *Bifidobacterium*, *Eubacterium_coprostanoligenes_group*, and *Rikenellaceae_RC9_gut_group* predominated in fecal samples, as shown in Figure 1E. Moreover, among the detected genera, *Escherichia-Shigella*, *Bacteroides*, *Prevotella* and *Ralstonia* predominated in milk samples, as shown in Figure 1F.

**Figure 2:**
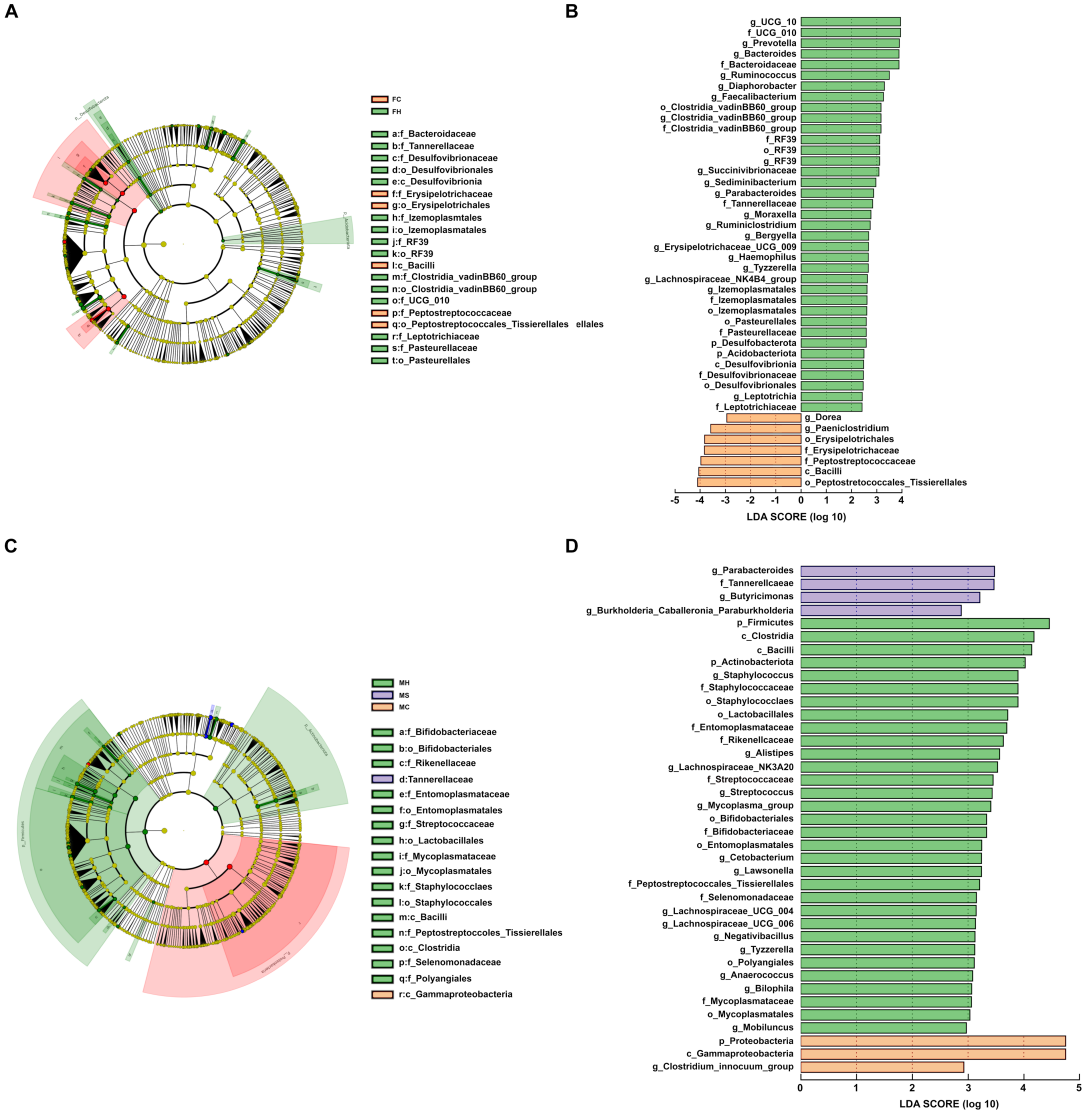
Cladogram shows linear discriminant analysis effect size (LEfSe) analysis of different microbiota among the three groups from phylum to genus level; linear discriminant analysis (LDA) score plot indicating the effects of different microbiota on difference among the three groups. **(A,B)** for feces; **(C,D)** for milk.

Referring to fecal samples, as shown in Figures 2A,B, none of the bacterial phyla showed significant differences among the three groups. Among the detected genera, 14 (average relative abundance >0.10%) were significantly different among three groups. In detail, *UCG-010*, *bacteroides*, *prevotella*, *ruminococcus*, *ralstonia*, *lachnospiraceae_NK4A136_group*, *clostridia vadinBB60_group*, *faecalibacterium*, *prevotellaceae UCG-004*, *parabacteroides*, *ruminiclostridium* and *sediminibacterium* were more abundant in the FC group, whereas *dgA-11 gut group* tended to be less abundant in the FC group compared to the other groups.

In terms of milk samples, as shown in Figures 2C,D, three of the bacterial phyla showed significant differences among the three groups (*p* < 0.05). Higher levels of *Firmicutes* and *Actinobacteriota* were registered in MH samples, while higher amounts of *Proteobacteria* were found in MC samples compared to the other two groups of samples. Among the detected genera, 11 (average relative abundance >0.10%) were significantly different among three groups. In detail, *Alistipes*, *Bifidobacterium*, *Streptococcus*, *Lawsonella*, *Lacnnospiraceae UCG-004*, *Fusicatenibacter*, *Bilophila* and *Anaerococcus* were more abundances in the MH group, whereas *Bilophila* and *[clostridium] Innocuum group* tended to be less abundant in the MH group compared with both other groups.

### 3.2 Fecal and milk metabolomic profiles among cows in different groups

**Figure 3:**
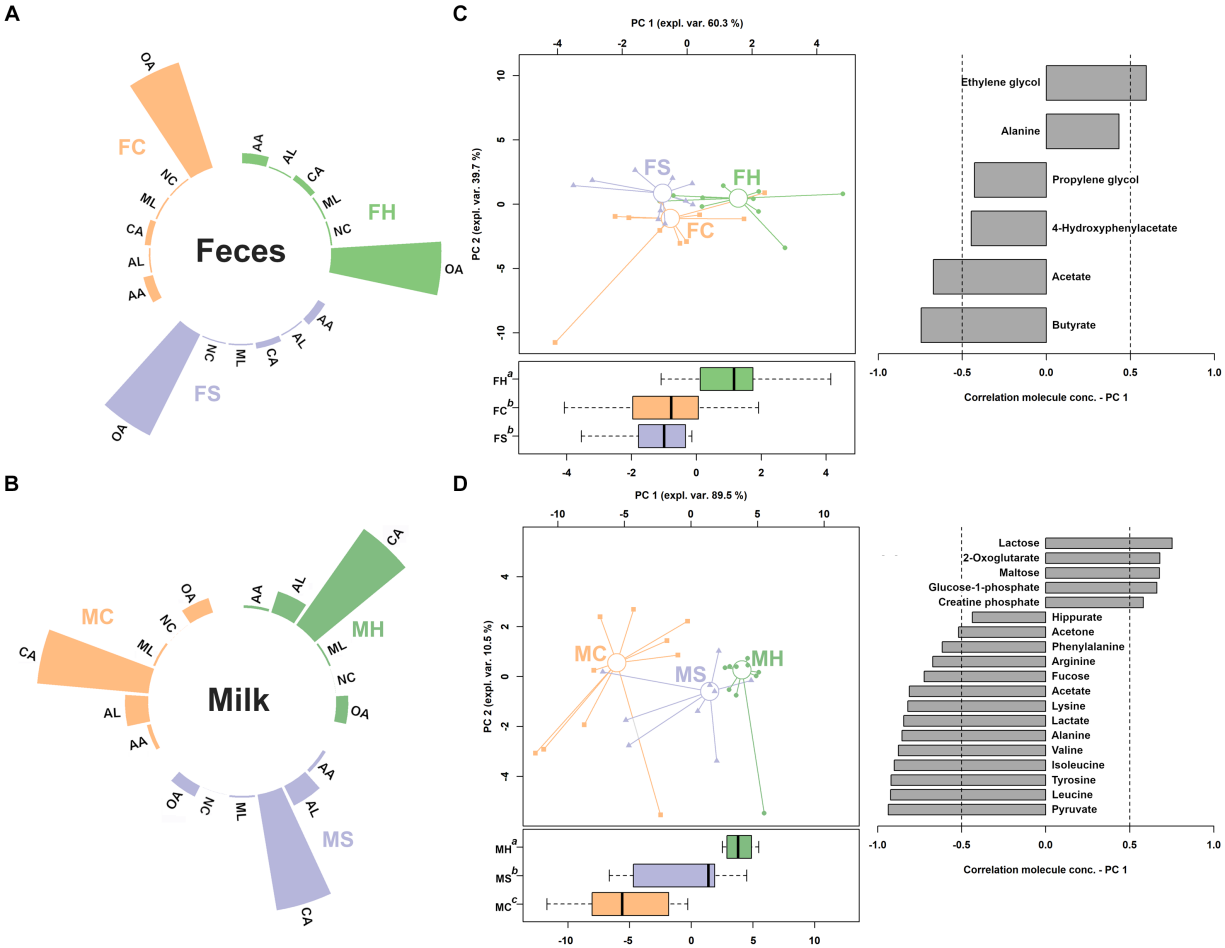
Relative abundance of the classes of molecules assigned in feces and milk metabolome **(A,B)**. CA, carbohydrates and derivatives; OA, organic acids and derivatives; AA, amino acids, peptides, and derivatives; NC, nucleosides, nucleotides, and analogues; AL, alcohols; ML, miscellaneous. **(C,D)** rPCA models for feces and milk, respectively.

Sixty-six and forty-six molecules were characterized by means of ^1^H-NMR in feces and milk samples, respectively. Their concentrations were provided in supporting materials. The characterized molecules could be sorted in six categories, namely carbohydrates and derivatives, organic acids and derivatives, amino acids, peptides, and derivatives, nucleosides, nucleotides, and analogues, alcohols, and miscellaneous Their relative concentrations were shown in Figures 3A,B. In fecal samples, eleven of the quantified molecules were significantly different among the three groups, namely glycine, O-acetylcholine, benzoate, glutamine, O-phosphocholine, tyrosine, creatine, methanol, valerate, acetoacetate, 1,3-dihydroxyacetone, pyruvate, propionate, acetate, 2,3-butanediol and ethanol. To gain a comprehensive insight into the trends exhibited by the identified molecules, their concentrations were utilized to construct a rPCA model, as depicted in Figure 2C. The first principal component (PC1) of its score plot, which encapsulates a substantial 60.3% of the total variability among the samples represented by the model, effectively captures the distinctions among samples from the three groups. Specifically, fecal samples obtained from cows experiencing clinical mastitis exhibited prominent features, characterized by elevated concentrations of propionate, acetate, pyruvate, acetoacetate, 2,3-butanediol, and ethanol. Conversely, lower concentrations were noted for glycine, O-acetylcholine, glutamine, O-phosphocholine, benzoate, tyrosine, and creatine.

Among the quantified molecules in milk samples, a total of twenty-eight exhibited significant variations across the three groups. Similar to feces samples, their concentrations were employed as a basis for an rPCA model, as shown in Figure 3D. PC1 of its score plot, which captures a substantial 89.5% of the overall sample variability as depicted by the model, effectively encapsulates the distinctions among samples from the three groups. In detail, milk from clinical mastitis cows was found to be mainly characterized by higher concentrations of lactose, 2-oxoglutarate, maltose, glucose—phosphate and creatine phosphate, and lower levels of hippurate, acetone, phenylalanine, arginine, fucose, acetate, lysine, lactate, alanine, valine, isoleucine, tyrosine, leucine and pyruvate.

**Figure 4:**
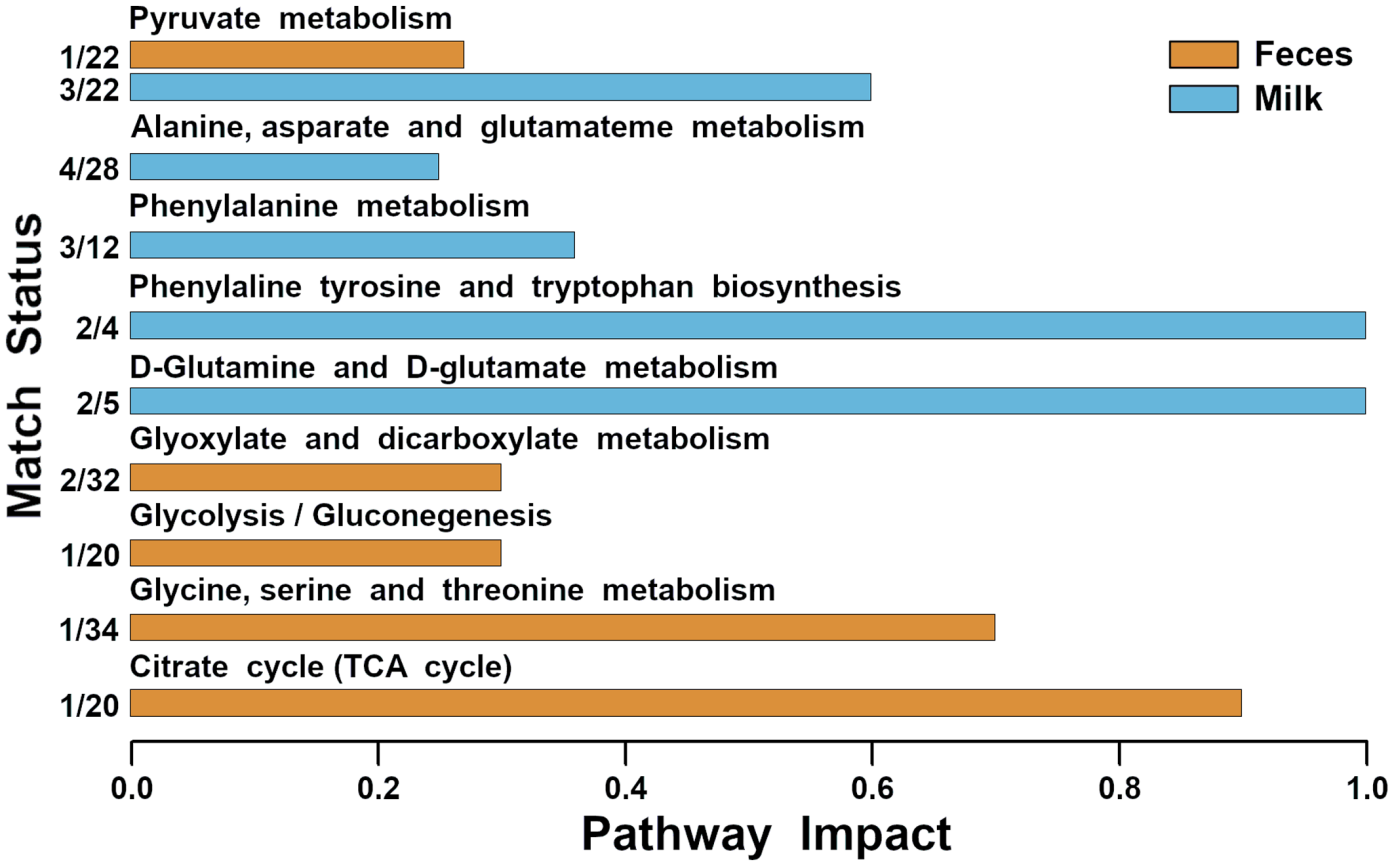
Metabolic pathways evidenced by enrichment analysis performed on the metabolites of feces and milk significantly different among the three groups (impact value >0.2).

Molecules that exhibited significant concentration changes among the three groups were selected as the foundation for a pathway enrichment analysis aimed at identifying the most pertinent pathways distinguishing these groups. This analysis revealed nine highlighted pathways: pyruvate metabolism, alanine, aspartate, and glutamate metabolism, phenylalanine metabolism, phenylalanine, tyrosine, and tryptophan biosynthesis, D-glutamine and D-glutamate metabolism, glyoxylate and dicarboxylate metabolism, glycolysis/gluconeogenesis, glycine, serine, and threonine metabolism, and the citrate cycle (depicted in Figure 4).

### 3.3 Network analysis between microbiome and metabolome

**Figure 5:**
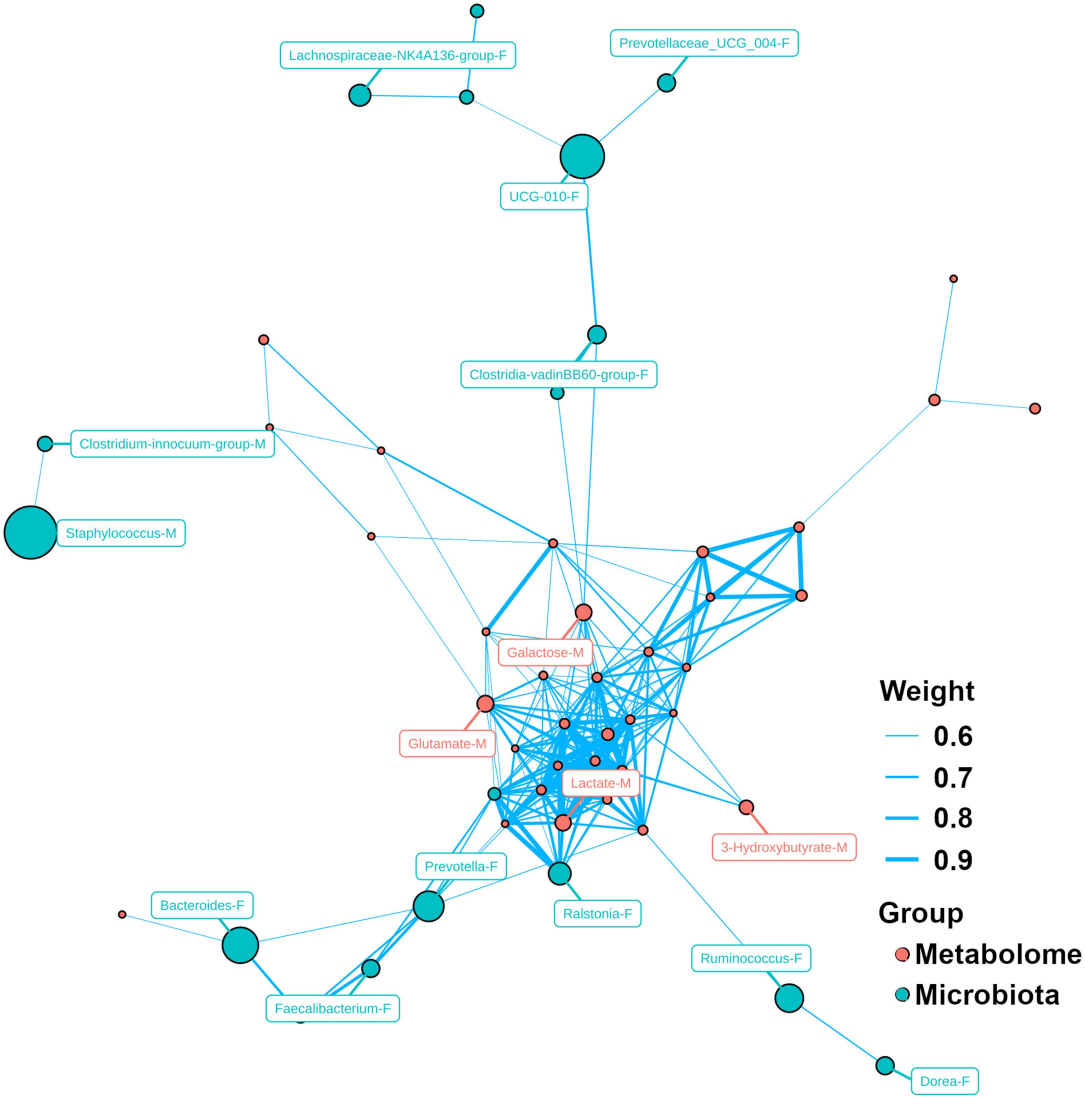
Network analysis for microbiome and metabolome. Capital letter (F and M) following by the name of bacteria/metabolite indicates the bacteria/molecule obtained from feces and milk, respectively.

Network analysis between microbiota and metabolites was performed, as shown in Figure 5. Metabolites play a central role in the network and there were correlations among their concentrations, such as glutamate, galactose, lactate and 3-hydroxybutyrate in milk. For microbiota, primarily the relative abundance of microbiota was correlated with each other. In milk, the relative abundance of *Staphylococcus* was correlated with that of *Clostridium_innocuum_group*. In feces, there was a correlation between the relative abundance of *Ruminococcus* and *Dorea*. Moreover, some correlations were also observed between the relative abundance of microbiota and the metabolite concentrations.

## 4 Discussion

In recent decades, the timely diagnosis and treatment of dairy cow mastitis have emerged as pivotal aspects within the dairy industry. Addressing this matter holds significant importance not only for farmers and processing industries but also for consumers. Such efforts contribute to heightened sensitivity and awareness concerning animal health and welfare across the entire dairy chain, in particular for public health threats posed by zoonotic bacteria (Halasa et al., 2007; Wang et al., 2023; Teng et al., 2024). Furthermore, the entero-mammary pathway theory proposes a potential connection between certain indigenous gut bacteria and the onset and progression of mastitis (Hu et al., 2020). Till now, the combination of microbiota and metabolomic analyses has exhibited the potential ability to further investigate the mechanism of mastitis. However, researchers have paid sufficient attention to the changes of milk metabolome and microbiota affected by mastitis (Luangwilai et al., 2021; Wang et al., 2022b), while only a few papers devoted their attention to the other biofluids, such as feces (Wang et al., 2022a), serum (Dervishi et al., 2018; Zhang et al., 2022), and urine (Zwierzchowski et al., 2020). Moreover, limited data exist concerning the assessment of correlations between the microbiota and metabolome of feces and milk influenced by mastitis, despite its proven potential to offer a comprehensive understanding of an animal’s response to both internal and external stimuli (Zhu et al., 2019; Eom et al., 2020; Kim et al., 2021). Addressing these gaps, this study represents the initial endeavor to concurrently profile the microbiota and metabolites present in both feces and milk obtained from the same dairy cows, with or without mastitis, through the application of both 16S rRNA and ^1^H-NMR techniques.

In our study, the community richness and diversity were significantly decreased in feces of cows with clinical mastitis compared to healthy and subclinically mstitic individuals (10 individuals/group), in agreement with Wang et al. (2022a). Moreover, the predominant bacteria were similar to previous studies (Bronzo et al., 2020), both in at the levels of phyla and genus. Within the dominant population of beneficial bacteria, a range of *Bacteroides* species can be identified. These species play a crucial role in the breakdown of polysaccharides and oligosaccharides, thereby furnishing essential nutrients and vitamins to the host and the wider community of intestinal microorganisms (Zafar and Saier, 2021).

In feces samples, the concentrations of eleven molecules showed significant differences among clinically mastitic, subclinically mastitic and healthy individuals. Among them, six molecules were highlighted by rPCA model, because their concentrations were significantly correlated with their importance in determining PC 1 orientation. They revealed higher levels of ethylene glycol and alanine and lower levels of propylene glycol, 4-hydroxyphenylacetate, acetate and butyrate in the feces of healthy dairy cows compared to the individuals with mastitis. Such results were in line with the findings of our previous study that mastitis could promote energy metabolism, while amino acids metabolism could be suppressed (Zhu et al., 2023). Propylene glycol undergoes conversion to propionate within the rumen, and subsequent absorption leads to its transformation into glucose within the liver. Recent investigations have reported that administering propylene glycol treatment to cows with subclinical ketosis yielded positive outcomes. These cows displayed an increased likelihood of recovery, reduced susceptibility to clinical ketosis and displaced abomasum, elevated milk yield during the initial lactation month, enhanced conception rates in their first service, and diminished risk of culling (Jenkins et al., 2015). Hoedemaker et al. found that peripartal propylene glycol supplementation could have positive effects on the metabolism of cows in a short-term period, referring to significantly decrease the concentrations of no esterified fatty acids and *β*-hydroxybutyrate, while concentrations of insulin-like growth factor (IGF)-I were significantly higher (Hoedemaker et al., 2004). This phenomenon has been corroborated by Hubner et al., who found that propylene glycol supplementation had improvements in health and milk production of cows with hypoglycemic (Hubner et al., 2022). Moreover, propylene glycol serves as a vital source for gluconeogenesis in ruminants and effectively curtails ketone formation. Administering propylene glycol to perinatal dairy cows represents an effective strategy to mitigate negative energy imbalances (Zhang et al., 2020). Furthermore, Lomander et al. revealed that early lactation-stage supplementation with propylene glycol led to heightened milk yield without subsequent metabolic compromise (Lomander et al., 2012). Hu et al. documented that cold exposure impacted the excretion levels of 4-hydroxyphenylacetate, a compound implicated in gut microbiome metabolism (Hu et al., 2021b). Notably, an elevated concentration of 4-hydroxyphenylacetate could potentially serve as a biomarker for predicting metritis risk in dairy cows (Dervishi et al., 2018). Intriguingly, all molecules displaying elevated levels in mastitic dairy cows are linked to energy metabolism. Nearly all dietary carbohydrates undergo fermentation to volatile fatty acids (acetate, propionate, and butyrate) within the rumen of dairy cows, with propionate being the predominant substrate for gluconeogenesis (Zhang et al., 2015). Additionally, evidence supports the migration of short-chain fatty acids (SCFAs) from the rumen to the mammary gland through an endogenous route (Hu et al., 2023). Pyruvate, a pivotal component in gluconeogenesis and the end product of glycolysis (Denton and Halestrap, 1979), holds significance as an intermediate metabolite for generating propionate from the succinic pathway or the lactate pathway (Jeyanathan et al., 2014). Conversion via the pyruvate dehydrogenase complex yields acetyl-CoA, which subsequently enters the TCA cycle. The TCA cycle plays a central role in cellular respiration and energy supply to living cells (Grassian et al., 2014), critically influencing cellular metabolic efficiency, cow metabolism, and production (Antunes-Fernandes et al., 2016). Within this context, our study suggests that mastitis-induced alterations in energy-related metabolic pathways contribute to adverse effects on milk production (Bravo and Wall, 2016). Furthermore, SCFAs possess the ability to activate aryl hydrocarbon receptor expression in intestinal epithelial cells, subsequently inducing IL-22 production and participating in the preservation of gut barrier integrity (Mudaliar et al., 2016; Li et al., 2022). Gut commensal microbes’ production of SCFAs has been demonstrated to enhance barrier integrity. Butyrate, by inhibiting HDAC and regulating the actin-associated protein synaptopodin, influences epithelial homeostasis (Wang et al., 2022a). Activation of PPARc by microbiota-derived butyrate also contributes to sustaining barrier integrity (Byndloss et al., 2017).

Compared to healthy animals, concentrations of nine molecules exhibited significant elevation in the milk of cows with both subclinical and clinical mastitis. In addition, the levels of another five molecules significantly increased only in the milk of cows with clinical mastitis, compared to that of healthy ones. These above molecules include hippurate, acetone, phenylalanine, arginine, fucose, acetate, lysine, lactate, alanine, valine, isoleucine, tyrosine, leucine, and pyruvate. Notably, eight out of the fourteen molecules belong to the chemical group of amino acids. This observation aligns with prior studies that have reported heightened concentrations of free amino acids, such as arginine, valine, and isoleucine, in milk samples with clinical mastitis (Mansor, 2012; Sundekilde et al., 2013). Statistically significant increases in milk amino acids could be attributed to augmented pathogen-specific fermentative processes and protein degradation activities (Ritter and Hanni, 1960). Pathway analysis results for milk corroborate this phenomenon. Among the various pathways involved, the phenylalanine, tyrosine, and tryptophan biosynthesis pathway emerges as particularly noteworthy. Phenylalanine, an indispensable amino acid, can be enzymatically converted into tyrosine with the assistance of phenylalanine hydroxylase and a biopterin cofactor (Brown, 1992). This molecule also serves as a precursor to catecholamines, neurotransmitters, and adrenaline-like substances (Waisbren et al., 2007). Tyrosine, an essential amino acid, is a fundamental constituent of numerous proteins, peptides, and enkephalins. It also functions as the precursor for hormones like catecholoestrogens and thyroxin (Lemmon and Schlessinger, 2010). Lactate, a significant end product of carbohydrate metabolism, can be generated by milk microorganisms or through anaerobic epithelial respiration in oxygen-deprived conditions following mastitis (Kneen and Annegarn, 1996). The presence of bacteria in milk has been associated with a distinctive metabolic fingerprint characterized by elevated lactate levels (Davis et al., 2004). In line with previous reports, the approximately 30-fold change in lactate further supports this finding (Davis et al., 2004).

Several molecules exhibited a declining trend from healthy to mastitis-affected cows. These molecules primarily provided insights into energy generation (lactose, maltose, 2-oxoglutarate, and glucose-1-phosphate) or protein digestion (creatine phosphate). These findings are in consonance with previous research, which documented lower concentrations of carbohydrates and energy-related metabolites, such as 2-oxoglutarate, in the milk of cows with clinical mastitis (Clara and Dvm, 2015). Pathway analyses undertaken in this study also substantiate this observation, with pyruvate metabolism and TCA cycle pathways highlighted. Collectively, our findings imply that clinical mastitis perturbs the metabolite equilibrium in cow milk by disrupting the TCA cycle within the mammary gland. N-acetylglucosamine, an amidic derivative of glucose and a secondary amide linking glucosamine and acetic acid, stems from amino sugar metabolism (Boudonck et al., 2009). Lactose is exocytosed by mammary epithelial cells and might also play a role in milk coagulation, thereby influencing milk quality, yield, and composition (Sundekilde et al., 2011). Reduced lactose content is observed in the milk of ruminants with mammary gland inflammation. The integrity of mammary cell membranes is compromised during clinical mastitis, allowing blood constituents to enter the milk (Bannerman, 2009). To maintain consistent osmotic pressure, lactose levels decrease accordingly.

## 5 Conclusion

In the current study, for the first time, we attempted to investigate differences in the microbiota profile and metabolite composition in feces and milk from healthy cows and from those with subclinical and clinical mastitis. Compared with those of healthy cows, the microbial community richness and diversity in the feces and milk of cows with clinical mastitis were significantly lower. The differences of feces microbiota were mainly evident at the level of genera. Mastitis caused also a strong disturbance in milk microbiota. Similarly, to microbiota profile, milk metabolome was affected by mastitis more deeply than fecal metabolome. Pathway analysis showed that amino acids metabolism and energy metabolism could be considered as the main pathways altered by mastitis. Further study would be benefited from enlarged sample size and the influence of animal host genetics would be considered due to their variated mastitis susceptibility, and microbial and metabolomic responses. Anyway, these findings would enrich the knowledge about the profiles of feces and milk microbiota and metabolites in cows with different udder health status, which might suggest new ways to prevent and treat mastitis, less reliant on drugs with potentially huge long-term side effects.

## Data availability statement

The original contributions presented in the study are publicly available. This data can be found in the NCBI Sequence Read Archive (SRA) database [Accession Number: PRJNA1095683].

## Ethics statement

The animal study was approved by the Animal Ethics Committee of Southwest Minzu University. The study was conducted in accordance with the local legislation and institutional requirements.

## Author contributions

CZ: Conceptualization, Data curation, Formal analysis, Investigation, Methodology, Writing – review & editing, Writing – original draft. YZ: Writing – original draft, Writing – review & editing. FY: Writing – review & editing, Writing – original draft, Supervision, Project administration, Methodology, Funding acquisition, Conceptualization. QZ: Writing – review & editing, Writing – original draft, Investigation, Formal analysis. XZ: Writing – review & editing, Writing – original draft. ZY: Writing – review & editing, Writing – original draft, Formal analysis. XD: Writing – review & editing, Writing – original draft. LL: Writing – review & editing, Writing – original draft, Methodology.

## Footnotes

^1^https://cloud.oebiotech.cn
