# Supplementary material for: Microbiome and metabolome analyses of milk and feces from dairy cows with healthy, subclinical, and clinical mastitis
Source: Front Microbiol. 2024 Jun 6;15:1374911. doi: 10.3389/fmicb.2024.1374911 (PMC11191547; doi:10.3389/fmicb.2024.1374911)
Supplement: Supplementary file 1 [file Table_1.DOCX]

Supplementary Material

# Supplementary Figures and Tables

## Supplementary Figures


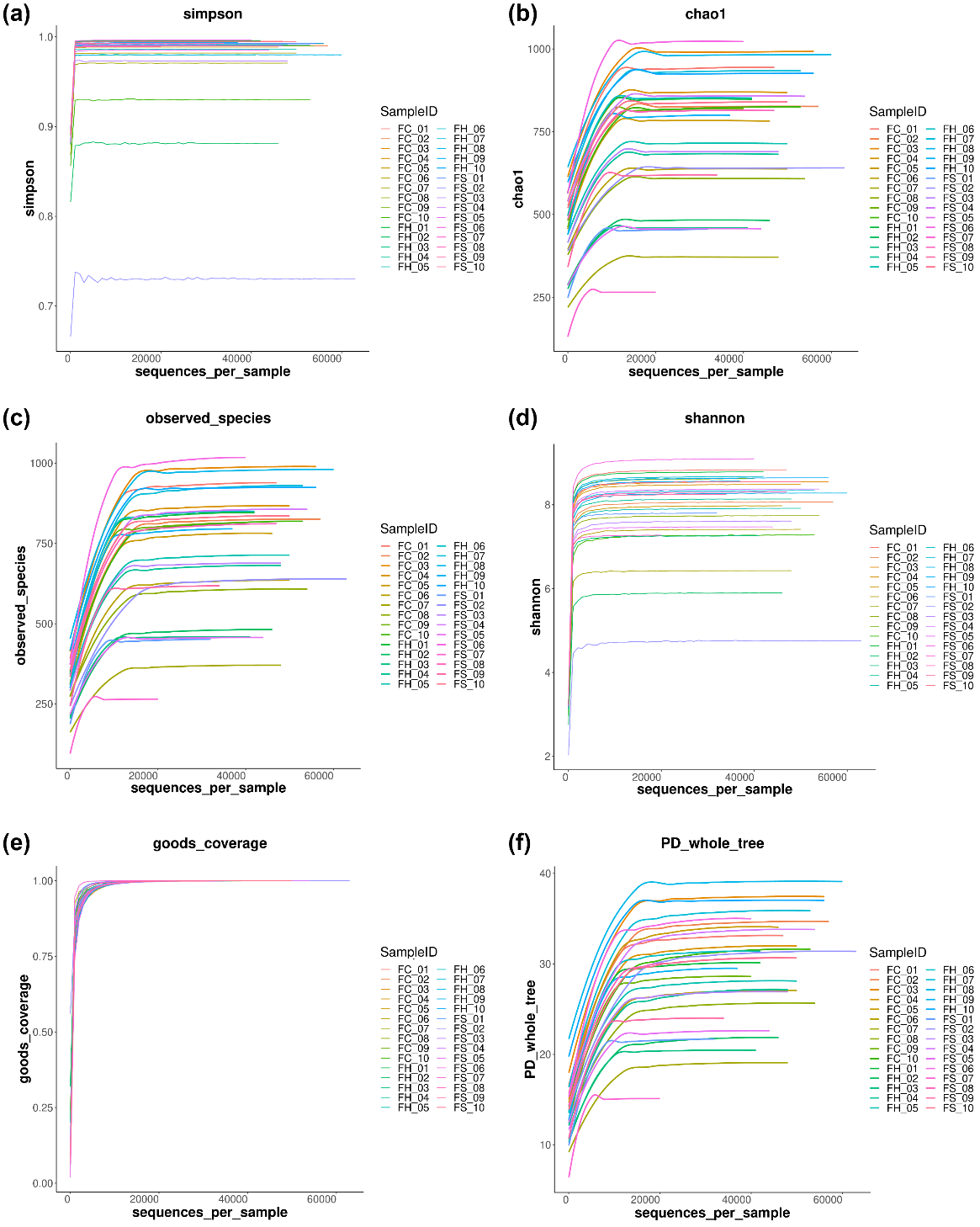


**Supplementary Figure 1.** The rarefaction curves of fecal microbiota based on the number of operational taxonomic units (OTUs) (n = 10). FH = healthy; FS = subclinical mastitis; FC = clinical mastitis.


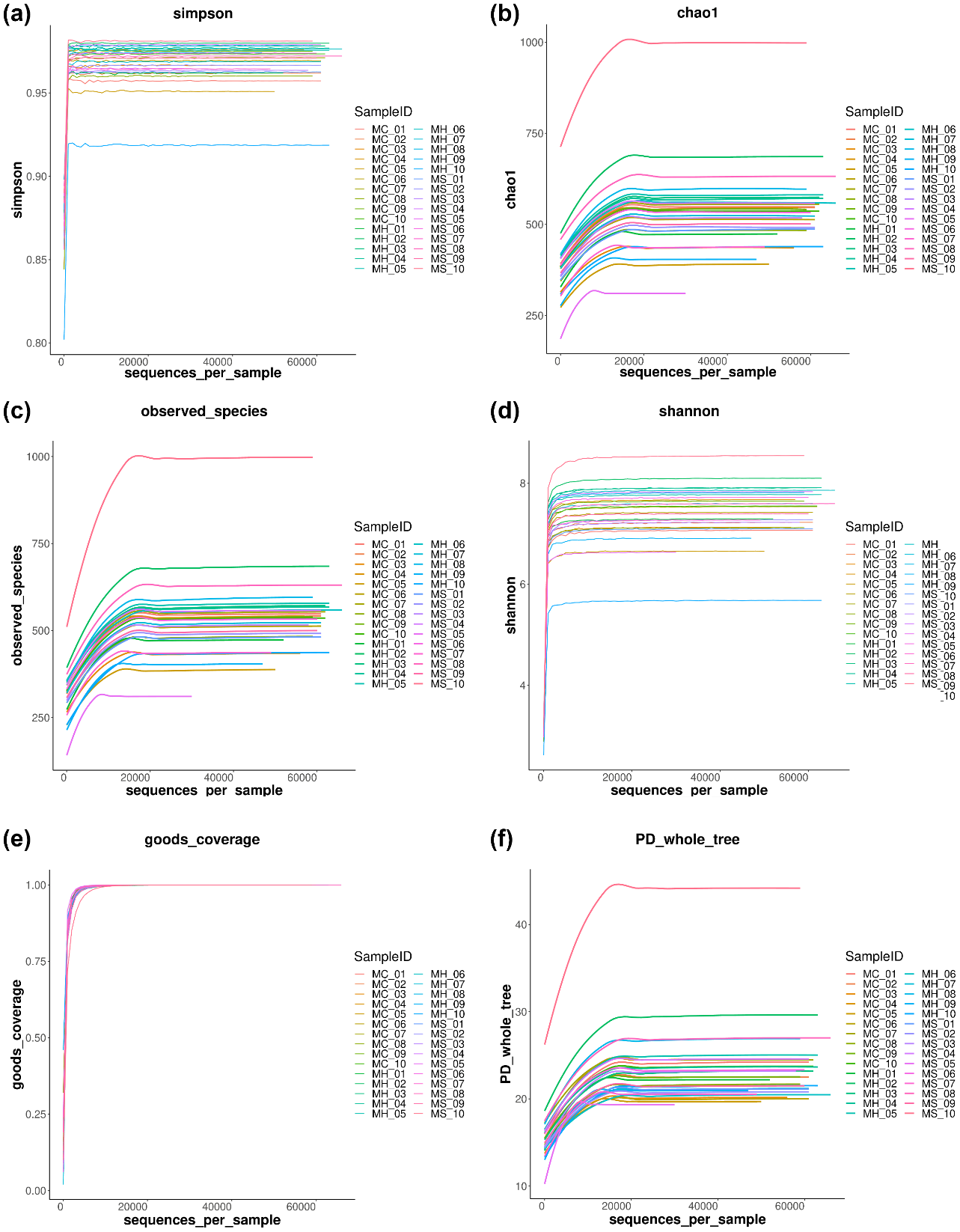


**Supplementary Figure 2.** The rarefaction curves of milk microbiota based on the number of operational taxonomic units (OTUs) (n = 10). FH = healthy; FS = subclinical mastitis; FC = clinical mastitis.





**Supplementary Figure 3.** ^1^H-NMR spectrum from one fecal sample representative of all the registered spectra. The name of each molecule appears over the signal used for its quantification. To ease the reader’s visual inspection, for each portion a spectrum with a convenient signal-to-noise ratio has been selected.





**Supplementary Figure 4.** ^1^H-NMR spectrum from one milk sample representative of all the registered spectra. The name of each molecule appears over the signal used for its quantification. To ease the reader’s visual inspection, for each portion a spectrum with a convenient signal-to-noise ratio has been selected.

## Supplementary Tables

**Supplementary Table 1.** Distribution of phylum in fecal samples

| NO. | Items | FH  (n=10) | FS  (n=10) | FC  (n=10) | SEM | *P*-value |
| --- | --- | --- | --- | --- | --- | --- |
| 1 | Firmicutes | 6.68×10^-1 a^ | 6.90×10^-1 a^ | 6.68×10^-1 a^ | 2.14×10^-2^ | 8.946×10^-1^ |
| 2 | Bacteroidota | 2.07×10^-1 a^ | 1.81×10^-1 a^ | 1.57×10^-1 a^ | 1.30×10^-2^ | 1.968×10^-1^ |
| 3 | Actinobacteriota | 3.53×10^-2 a^ | 6.72×10^-2 a^ | 1.14×10^-1 a^ | 2.17×10^-2^ | 9.026×10^-1^ |
| 4 | Proteobacteria | 5.09×10^-2 a^ | 2.40×10^-2 a^ | 2.26×10^-2 a^ | 8.78×10^-3^ | 7.393×10^-1^ |
| 5 | Spirochaetota | 3.54×10^-2 a^ | 3.67×10^-2 a^ | 1.95×10^-2 a^ | 7.87×10^-3^ | 2.651×10^-1^ |
| 6 | Fusobacteriota | 7.42×10^-4 a^ | 1.33×10^-4 a^ | 1.86×10^-2 a^ | 6.15×10^-3^ | 4.714×10^-1^ |
| 7 | Other | 2.74×10^-4 a^ | 1.44×10^-4 a^ | 2.53×10^-5 a^ | 5.94×10^-5^ | 2.384×10^-1^ |
